# Survival predictors of metastatic angiosarcomas: a surveillance, epidemiology, and end results program population-based retrospective study

**DOI:** 10.1186/s12885-020-07300-7

**Published:** 2020-08-18

**Authors:** Shihong Ren, Yucheng Wang, Zhan Wang, Jinxiang Shao, Zhaoming Ye

**Affiliations:** 1 Department of Orthopaedics, Centre for Orthopaedic Research, Orthopedics Research Institute of Zhejiang University, The Second Affiliated Hospital, Zhejiang University School of Medicine, 88 Jiefang Road, Hangzhou, Zhejiang 310000 P.R. China; 2 Department of Orthopedics, The First People’s Hospital of Wenling, No. 333, Chuanannan Road, Chengxi Street, Wenling, 317500 P.R. China; 3 Hebei North University, Zhangjiakou, 075000 P.R. China

**Keywords:** Angiosarcoma, Survival predictors, Overall survival, SEER, Retrospective study, Treatment

## Abstract

**Background:**

Angiosarcomas (AS) have poor prognosis and often metastasize to distant sites. The potential predictors of metastatic angiosarcomas (MAS) have not been extensively investigated. The main objective of this study was to identify survival predictors of MAS.

**Methods:**

Surveillance, Epidemiology, and End Results (SEER) datasets were used to identify patients with MAS from 2010 to 2016. Risk predictors were determined with the aid of Kaplan-Meier and Cox regression model analyses.

**Results:**

A total of 284 MAS patients met the study entry criteria. Among these, 121 patients (42.6%) were diagnosed with metastasis in bone, 26 in brain (9.2%), 86 in liver (30.3%) and 171 in lung (60.2%). Overall, 96 patients (33.8%) had two or more metastatic sites. The 1- and 3-year overall survival (OS) rates were 20.8 and 3.8% while 1- and 3-year cancer-specific survival (CSS) rates were 22.0 and 5.2%, respectively. Cox regression analysis revealed chemotherapy, radiation treatment (RT) and tumor size ≤10 cm as independent favorable predictors of OS. In terms of CSS, tumor grade IV, tumor size > 10 cm and absence of chemotherapy were independent adverse predictors. Surgery did not prolong survival outcomes (both OS and CSS) in the current cohort.

**Conclusion:**

MAS is associated with extremely poor survival. Chemotherapy, RT, and tumor size are independent predictors of OS. Chemotherapy and tumor size are independent prognostic factors of CSS. Chemotherapy is therefore recommended as the preferred treatment option for MAS patients.

## Background

Angiosarcomas (AS) are rare, highly malignant soft-tissue sarcomas of vascular or lymphatic origin, which account for approximately 1–2% of all soft tissue sarcomas [1, 2]. These sarcomas can develop in any anatomic location of the body [1], frequently manifesting as cutaneous disease in elderly men [3]. While the incidence of AS continues to increase [4–6], treatment is challenging and prognosis remains poor, with overall survival (OS) ranging from 30 to 50 months [7, 8] and 5-year survival rates between 10 and 50% [3, 8–10]. Compared with localized disease, metastatic angiosarcomas (MAS) patients present significantly shorter median OS (3 months) [11], ultimately succumbing to metastatic disease [12]. The majority of published studies to date, predominantly case series and individual institution analyses, have analyzed outcomes and prognosis for localized AS [13–17]. Here, we have conducted a retrospective population-based cohort study on patients selected from the SEER database, with a view to delineating the predictors of MAS.

## Methods

### Patients

Patient data (from 2010 to 2016) were accessed from the Surveillance, Epidemiology, and End Results (SEER)18 registry of the National Cancer Institute, an authoritative cancer research center that uses hospital registry data accredited by the Commission on Cancer. Use of SEER data does not require approval by the Institutional Review Board.

SEER*Stat (Version 8.3.6) was applied to identify patients diagnosed with angiosarcoma using the International Classification of Diseases for Oncology 3rd Edition morphological code (9120). All patients met the entry criteria based on positive histological findings. Thirteen patients diagnosed with positive exfoliative cytology, radiography or unknown histology were excluded, along with one patient for whom the mode of therapy and survival months were unknown. Statistical variables included age at diagnosis (≤ and > 60 years), gender (female and male), race (white, black, and other), year of diagnosis (2010–2012, 2013–2014, and 2015–2016), tumor grade (I + II, III, IV, and unknown), tumor size (≤ or > 10 cm, and unknown), chemotherapy, radiation treatment (RT), surgery treatment (ST) and vital status (dead and alive). Metastatic sites were divided into two categories according to number, specifically, 1 and ≥ 2 sites.

### Statistical analysis

Statistical analyses were performed using Microsoft Excel 2019 (Microsoft Corp., Redmond, WA, USA) and SPSS statistics (version 25.0, IBM corp., USA). Overall survival (OS) was defined as time from diagnosis to death induced by any cause or last follow-up. Cancer-specific survival (CSS) was regarded as time from diagnosis to death specifically due to AS. Observations were censored if patients were alive at the time of last follow-up. Survival curves were constructed using GraphPad Prism 8 software (La Jolla, California). The Kaplan–Meier method was applied to calculate survival rates and median survival. We also used Kaplan–Meier method to perform univariate analysis. Statistical significance was calculated with the log-rank test. Variables of *P* < 0.05 in univariate analyses were included for multivariate analyses. Independent predictors of OS and CSS were determined using the multivariate Cox regression model. We used ‘Enter’ method in Cox regression. Hazard ratios (HR) and 95% confidence intervals (CI) were employed to determine the effects of various factors on OS and CSS. Differences were considered statistically significant at *P* < 0.05.

## Results

### Patients and tumor characteristics

From 2010 to 2016, 284 patients with MAS were identified from the SEER database. Descriptive statistics are presented in Table 1 and Table S1. Median survival rates in relation to different variables are listed in Table 2 and Table S2. Patients presented with MAS at a median age of 63 years (range, 0–95 years). In total, 156 patients (54.9%) were > 60 years and 128 (45.1%) were ≤ 60 years of age. MAS affected men (182, 64.1%) more frequently than women (102, 35.9%), consistent with previous results [18–20]. The majority of patients (80.3%) were white, similar to earlier reports [1, 21, 22]. In terms of year of diagnosis, 111 (39.1%) patients were diagnosed between 2010 and 2012, 86 (30.3%) between 2013 and 2014, and 87 (30.6%) between 2015 and 2016. Regarding metastatic sites, 121 patients (42.6%) were diagnosed with metastases in bone, 26 (9.2%) in brain, and 86 (30.3%) in liver. Lung (171, 60.2%) was identified as the most frequent site of metastasis, consistent with published data [13, 23, 24]. Two or more metastatic sites were identified in 96 patients (33.8%). Histologically, 6 cases (2.1%) were grade I + II, 52 (18.3%) were grade III, and 50 (17.6%) were grade IV. More than half the tumors (176, 62.0%) were of unknown grade. Overall, 110 (38.7%) tumors were ≤ 10 cm, 44 (15.5%) were > 10 cm, and sizes were unknown for approximately half of the tumors. More than half (51.1%) of the patients received chemotherapy, 66 (23.2%) received RT, and 83 (29.2%) received ST. A total of 253 (89.1%) deaths were recorded, 180 of which were attributed to MAS-related mortality. In terms of primary tumor sites, 35(12.3%) cases occurred in head and neck, 112(39.4%) in visceral/ deep soft tissue, 47(16.5%) in trunk and limbs, 90(31.7%) in other sites. The 1- and 3-year OS and CSS rates for the entire cohort were 20.8 and 3.8% and 22.0 and 5.2%, respectively.

**Table 1:** Demographics of 284 patients with metastatic angiosarcomas identified from SEER database between 2010 and 2016

Category	N (%)
**Age at diagnosis(years)**	
≤ 60	128(45.1%)
> 60	156(54.9%)
mean	60.5 Years
median	63 Years
**Gender**	
Female	102(35.9%)
Male	182(64.1%)
**Race**	
White	228(80.3%)
Black	25(8.8%)
Other	31(10.9%)
**Year of diagnosis**	
2010–2012	111(39.1%)
2013–2014	86(30.3%)
2015–2016	87(30.6%)
**Metastatic sites at diagnosis**	
Bone	
Yes	121(42.6%)
No	163(57.4%)
Brain	
Yes	26(9.2%)
No	258(90.8%)
Liver	
Yes	86(30.3%)
No	198(69.7)
Lung	
Yes	171(60.2%)
No	113(39.8%)
**Number of metastatic sites**	
1	188(66.2%)
≥ 2	96(33.8%)
**Grade**	
I + II	6(2.1%)
III	52(18.3%)
IV	50(17.6%)
Unknown	176(62.0%)
**Size(cm)**	
≤ 10	110(38.7%)
> 10	44(15.5%)
Unknown	130(45.8%)
**Treatment**	
Chemotherapy	
Yes	145(51.1%)
No	139(48.9%)
RT	
Yes	66(23.2%)
No	218(76.8%)
ST	
Yes	83(29.2%)
No	201(70.8%)
**Dead**	
Yes	253(89.1%)
No	31(10.9%)
**1-year OS rate**	20.8%
**3-year OS rate**	3.8%
**1-year CSS rate**	22.0%
**3-year CSS rate**	5.2%

*Abbreviations*: *SEER* Surveillance, Epidemiology, and End Results, *OS* overall survival, *CSS* cancer-specific survival, *RT* radiation treatment, *ST* surgery treatment

**Table 2:** Median survival data (months) of metastatic angiosarcomas

Variable	OS		CSS	
	Estimate ± SE	95%CI	Estimate ± SE	95%CI
**Age at diagnosis (years)**				
≤ 60	5.0 ± 0.9	3.323–6.677	5.0 ± 0.9	3.228–6.772
> 60	2.0 ± 0.4	1.168–2.832	2.0 ± 0.5	1.005–2.995
**Gender**				
Female	4.0 ± 0.7	2.575–5.425	4.0 ± 1.0	2.100–5.900
Male	3.0 ± 0.6	1.795–4.205	3.0 ± 0.6	1.845–4.155
**Race**				
White	3.0 ± 0.5	2.063–3.937	3.0 ± 0.5	1.972–4.028
Black	4.0 ± 1.7	0.757–7.243	4.0 ± 1.4	1.309–6.691
Other	6.0 ± 4.2	0.000–14.305	6.0 ± 2.6	0.842–11.158
**Year of diagnosis**				
2010–2012	4.0 ± 1.0	2.068–5.932	3.0 ± 0.9	1.280–4.720
2013–2014	4.0 ± 0.8	2.403–5.597	3.0 ± 0.9	1.272–4.728
2015–2016	3.0 ± 0.6	1.844–4.156	3.0 ± 1.0	1.124–4.876
**Grade**				
I + II	N/A	N/A	3.0 ± 0.6	1.863–4.137
III	4.0 ± 1.0	2.137–5.863	4.0 ± 1.5	0.986–7.014
IV	4.0 ± 0.7	2.534–5.466	4.0 ± 0.7	2.588–5.412
**NO. of metastatic sites**				
1	3.0 ± 0.6	1.789–4.211	4.0 ± 0.9	2.162–5.838
≥ 2	3.0 ± 0.7	1.589–4.411	3.0 ± 0.7	1.629–4.371
**Size (cm)**				
≤ 10	6.0 ± 1.1	3.887–8.113	9.0 ± 1.9	5.337–12.663
> 10	2.0 ± 0.7	0.701–3.299	3.0 ± 0.6	1.840–4.160
**Treatment**				
Chemotherapy				
Yes	8.0 ± 1.0	6.083–9.917	8.0 ± 1.3	5.444–10.556
No	1.0 ± 0.2	0.692–1.308	1.0 ± 0.2	0.636–1.364
**RT**				
Yes	7.0 ± 1.6	3.875–10.125	6.0 ± 1.6	2.870–9.130
No	3.0 ± 0.4	2.202–3.798	3.0 ± 0.4	2.200–3.800
**ST**				
Yes	3.0 ± 0.5	2.022–3.978	3.0 ± 0.6	1.876–4.124
No	3.0 ± 0.7	1.710–4.290	3.0 ± 0.7	1.621–4.379
**Overall**	3.0 ± 0.5	2.078–3.922	3.0 ± 0.5	1.979–4.021

*Abbreviations*: *OS* overall survival, *CSS* cancer-specific survival, *N/A* means that the median survival time was not available due to death event occurring in fewer than 50% of cases in the cohort, *SE* standard error, *CI* confidence interval, *RT* radiation treatment, *ST* surgery treatment

### Univariate analysis of variables associated with OS or CSS in MAS patients

**Fig. 1:**
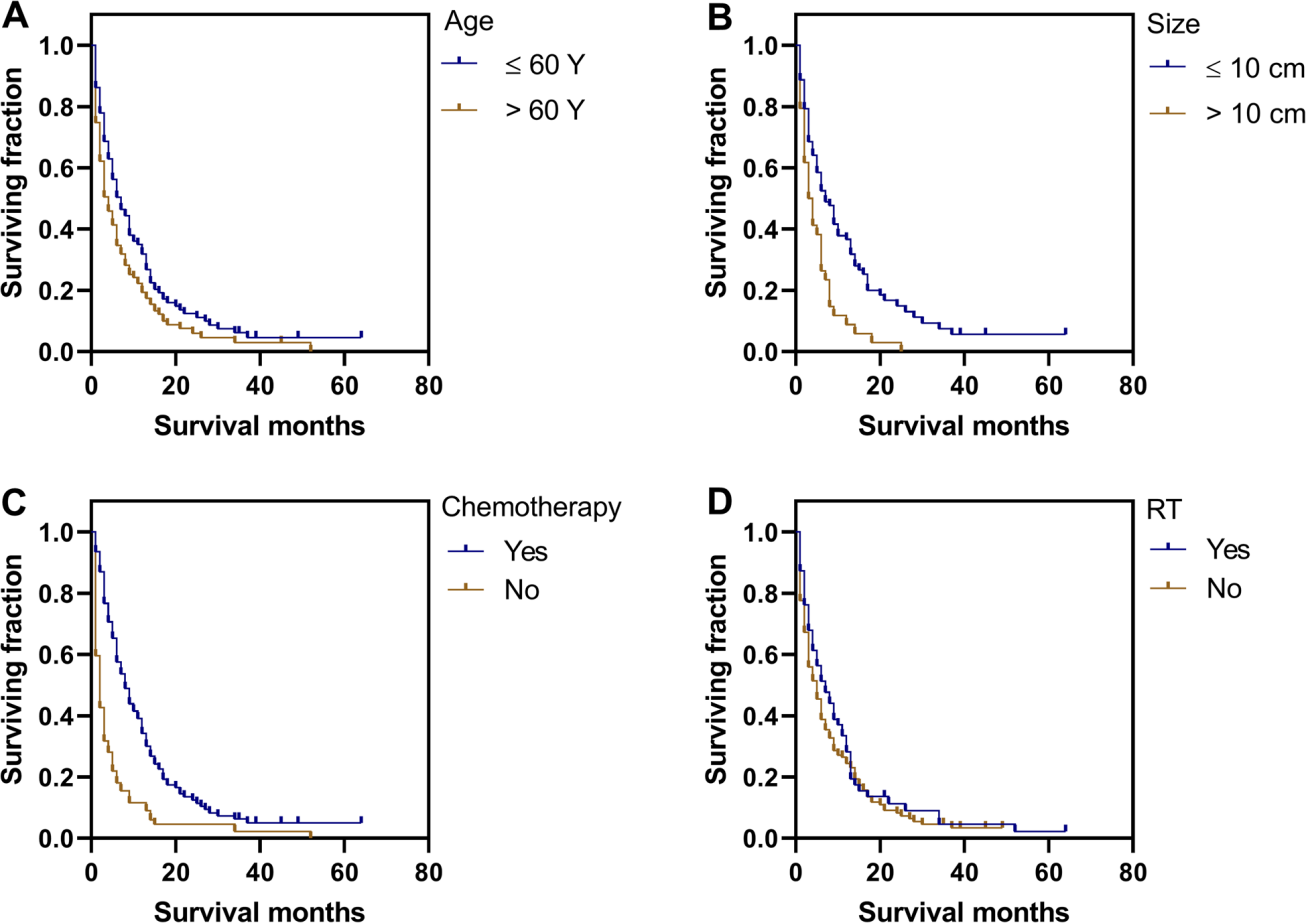
Kaplan-Meier method estimated OS in patients with metastatic angiosarcomas stratified by **a** age at diagnosis (years), **b** size, **c** chemotherapy, **d** RT, radiation treatment

**Fig. 2:**
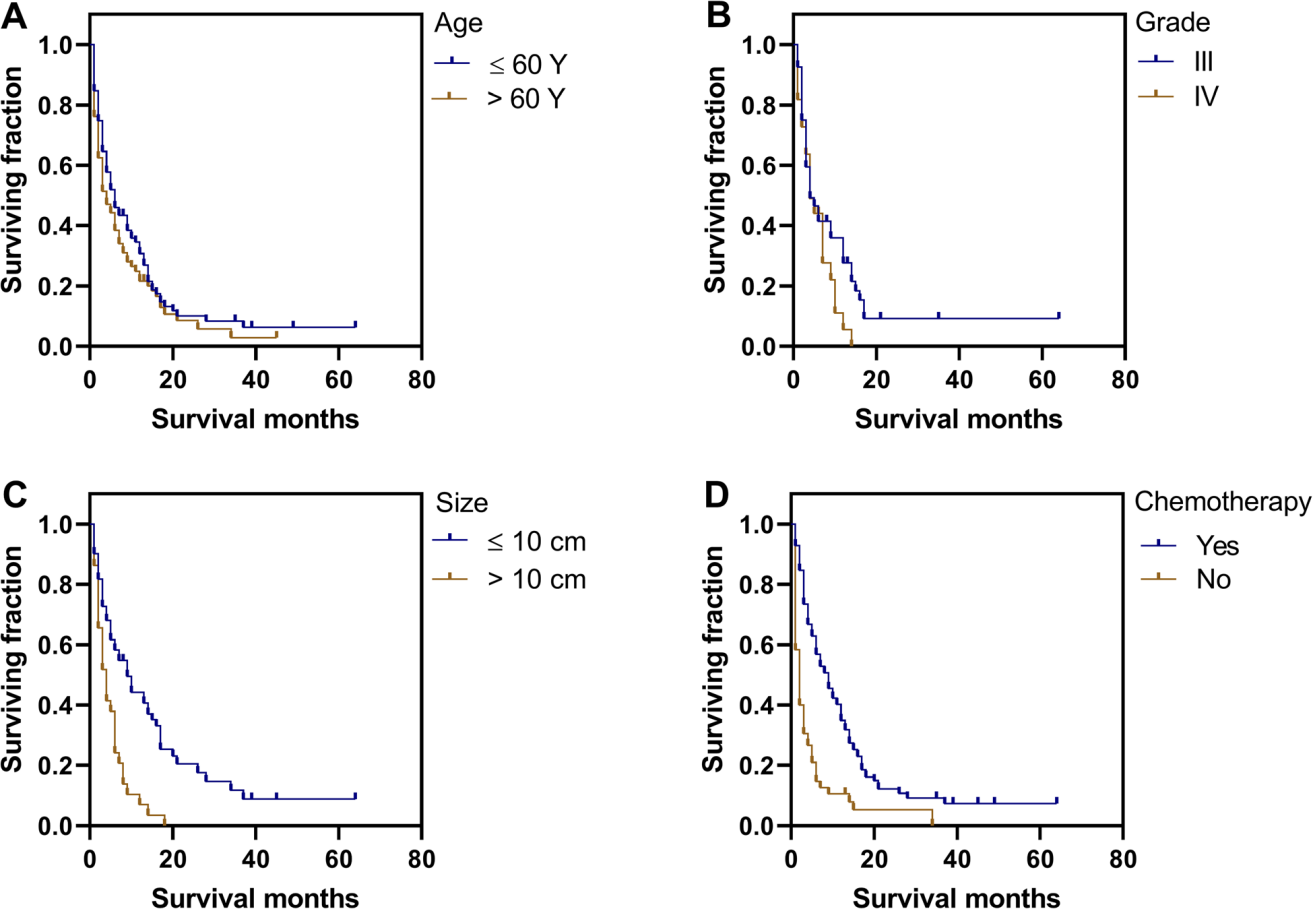
Kaplan-Meier method estimated CSS in patients with metastatic angiosarcomas stratified by **a** age at diagnosis (years), **b** grade, **c** size, **d** chemotherapy

Univariate analysis using the log-rank test was conducted to analyze potential prognostic factors (Table 3 and Table S3). Our tests revealed that age > 60 years was significantly associated with poorer OS (*P* = 0.003, Table 3, Fig. 1a) and CSS (*P* = 0.036, Table 3, Fig. 2a). Neither gender nor race was significantly associated with OS or CSS. Similarly, year of diagnosis and number of metastatic sites were not predictors of OS and CSS. Patients with grade IV tumors had poorer CSS (*P* = 0.024, Table 3, Fig. 2b), but not OS. Notably, smaller tumor size (≤10 cm) was a beneficial predictor for both OS (*P* < 0.001, Table 3, Fig. 1b) and CSS (*P* < 0.001, Table 3, Fig. 2c). Significant differences in both OS (*P* < 0.001) and CSS (*P* < 0.001) were observed between chemotherapy-administered and non-administered groups (Table 3, Figs. 1c and 2d). Patients receiving RT showed better OS (*P* = 0.009, Table 3, Fig. 1d), but not CSS. In the current cohort, surgery did not prolong the survival times of patients in terms of both OS (*P* = 0.192) and CSS (*P* = 0.251). Regarding survival rates of different primary tumor sites, compared with visceral/deep soft tissue in OS, patients of head and neck tumors had better survival (*P* = 0.038, Table S3), while the comparison of entire cohort did not reach striking disparities (OS: *P* = 0.162, CSS: *P* = 0.667, Table S3).

**Table 3:** Kaplan–Meier method performs univariate analysis of variables for OS and CSS in patients of metastatic angiosarcomas

Category	OS (log-rank *P*-value)	CSS (log-rank *P*-value)
**Age at diagnosis(years)**	0.003	0.036
**Gender**	0.567	0.139
**Race**	0.594	0.348
**Year of diagnosis**	0.770	0.955
**Grade** (III vs. IV)	0.187	0.024
**Number of metastatic sites**	0.287	0.113
**Size** (≤10 cm vs. > 10 cm)	< 0.001	< 0.001
Treatment		
Chemotherapy	< 0.001	< 0.001
RT	0.009	0.077
ST	0.192	0.251

*Abbreviations*: *OS* overall survival, *CSS* cancer-specific survival, *RT* radiation treatment, *ST* surgery treatment

### Multivariate analysis of independent predictors of OS or CSS in MAS patients

Age at diagnosis (years), size (≤10 cm vs. > 10 cm), chemotherapy and RT were included for multivariate analysis of OS. Age at diagnosis(years), grade, size (≤10 cm vs. > 10 cm), chemotherapy were included for multivariate analysis of CSS. The results of multivariate analyses for the entire cohort are shown in Table 4. Size, chemotherapy and RT were significant independent predictors of OS. Size, grade and chemotherapy were significant independent predictors of CSS.

**Table 4:** Cox proportional hazards model performs multivariate analysis for OS and CSS in metastatic angiosarcomas

Category	OS		CSS	
	HR (95%CI)	***P***-value	HR (95%CI)	***p***-value
**Age at diagnosis(years)**	1.074(0.824–1.400)	0.597	1.160(0.854–1.575)	0.342
**Grade** (III vs. IV)	–	–	2.052(1.220–3.452)	0.007
**Size** (≤10 cm vs. > 10 cm)	1.956(1.345–2.843)	< 0.001	2.316(1.495–3.586)	< 0.001
**Chemotherapy**	2.956(2.233–3.913)	< 0.001	3.308(2.379–4.600)	< 0.001
**RT**	1.580(1.162–2.149)	0.004	–	–

*Abbreviations*: *OS* overall survival, *CSS* cancer-specific survival, *HR* hazards ratio, *RT* radiation treatment, *CI* confidence interval

## Discussion

To date, there is no definitive treatment guideline for MAS. Patients with this cancer have a poor prognosis [8, 11, 25]. Therefore, it is necessary to evaluate the risk factors for this disease. In this series, patients with tumor size ≤10 cm, receipt of RT and chemotherapy had better OS. While patients with tumor size > 10 cm, grade IV tumors and absence of chemotherapy had poorer CSS. The current study could aid the optimum of therapeutic regimens for these patients.

### Survival by age

Two earlier reports which analyzed AS revealed a negative impact of older age on survival [3, 10], conflicting with other two investigations [11, 22]. The relationship between survival and age at diagnosis in patients with MAS has not been extensively explored until now. In our study, multivariate analyses showed that age was not a significant independent predictor. Our current findings are potentially attributable to the significantly short median survival time (3 months) of MAS patients, which may not reflect the relationship between the two.

### Survival by gender, race, year of diagnosis, number of metastatic sites and primary tumor sites

Our current findings were consistent with prior studies showing that gender [21], race, and year of diagnosis do not affect OS of AS patients [3, 11, 19]. Limited studies to date have focused on the relationship between number of metastatic sites and MAS survival. In the current series, compared to patients with ≥2 metastatic sites, we observed no improvement in OS of patients with one metastatic site and a slight benefit in CSS, which did not reach statistical significance. Regarding the relationship between the primary tumor sites and patients’ survival time, patients of head and neck disease had better median survival. However, the difference was not statistically significant. Maybe tumor metastasis is one of the major causes of death in these patients. So, early detection is very important for prolonging patients’ survival time.

### Survival by grade

The results of the earlier studies regarding the relation between tumor grade and patients’ survival time varied. A number of previous studies have documented no association of tumor grade with OS of AS [7, 26]. Conversely, Kathryn et al. [20] reported that higher tumor grade is predictive of greater risk of death in primary mediastinal sarcoma, conflicting with the conclusion reached by Brett et al. and Manjari et al. [21, 27]. In the present study, patients with tumor grade IV showed poorer CSS, compared with the grade III group. However, the underlying reason remains to be explained and our results require further validation.

### Survival by tumor size

Two earlier studies reported that larger tumor size does not impair OS of AS [3, 20]. In contrast, a pooled analysis incorporating 75 articles involving 186 patients suggested that tumor size (< 10 cm) was the only significant favorable factor for OS of hepatic AS in adults [28]. Several other documents presented tumor size was a critical predictor [7, 10, 18, 26, 29]. Data from our large-scale investigation revealed that tumor size was an independent prognostic factor for this rare disease. Larger tumor size may have a longer course of disease and earlier metastasis, leading to poorer survival than cases of MAS with tumors ≤10 cm. Despite the conflicting results in the literature, we believe that tumor size is a vital predictor of survival in MAS.

### Survival by treatment type (chemotherapy, RT, and ST)

Treatment results for AS vary significantly. An earlier retrospective analysis of postoperative AS led to the conclusion that chemotherapy dose not confer an OS benefit [21]. Consistently, a single-institution investigation including 88 patients with cutaneous AS revealed no clear benefits of chemotherapy on OS [22]. Conversely, Young et al. reported that chemotherapy should be used as the primary treatment option for MAS [1]. A phase II trial including patients with metastatic or unresectable AS supported the therapeutic efficacy of paclitaxel [14]. Many published data similarly suggest that chemotherapy is associated with improved OS in AS [12, 13, 30, 31]. In our study, multivariate analysis identified chemotherapy as a significantly independent variable of prolonged survival. Maybe, discrepancies of epithelioid component [8] in different tumor stage cause the aforementioned conflicting results.

Conic et al. analyzed the outcome of cutaneous AS patients and disclosed no significant impacts of RT on OS [3], same results were confirmed by Buehler et al. and Zhang et al. [11, 26]. In contrast, Ogawa et al. [19] reported that RT effectively improved OS in 48 patients with localized AS of the scalp and face. In the current series, RT induced a significant improvement in OS. In terms of CSS, better median survival was observed in patients treated with RT relative to median survival in the absence of RT. However, the difference was not statistically significant. Our results require further validation. In addition, due to the effect of RT causing development of sarcomas [6], it should be used cautiously in treatment of AS.

A retrospective study disclosed no significant impacts of ST on OS for patients presented with metastatic disease [11], consistent with the current findings. This may be due to the fact that ST can only be applied for resection of localized or regional lesions, but does not improve the overall condition of MAS patient. Conversely, Abraham and colleagues analyzed 82 patients from one institution and confirmed that aggressive ST enhanced long-term survival in the majority of patients [24]. Patients with non-metastatic scalp AS subjected to ST showed a subsequent improvement in OS [10]. Different responses to treatment methods may result from various underlying diseases of AS patients and tumor heterogeneity. However, the intrinsic causes need to be further investigated.

Our study has several limitations that should be considered. First, the available information was incomplete due to the retrospective nature of the investigation. Prospective studies should be conducted to verify our conclusions. Second, the SEER database does not provide other important information, such as detailed radiotherapy and chemotherapy regimens, basic health status of patients, and surgical protocols, which may cause bias of results. Further research should focus on inclusion of these variables to provide supplementary information. Third, imbalance ratios of variables (for instance, RT, yes:no = 66:218), may contribute to results bias in this study. Despite these limitations, we successfully analyzed the predictors and outcomes of MAS for the first time. In addition, the SEER database collects tumor information based on highly unified standards from multiple centers, providing the largest quantity of tumor data, especially for rare tumors.

## Conclusions

In this study, we analyzed the survival predictors of MAS, known for its extremely poor survival rates, in 284 patients. Chemotherapy, RT and tumor size ≤10 cm were identified as independent protective predictors of OS. Chemotherapy and tumor size ≤10 cm were independent favorable prognostic factors of CSS. Grade IV was associated with poorer survival of CSS. The number of metastatic sites did not appear to affect OS and CSS. Based on the collective findings, we recommend chemotherapy as the preferred treatment option for MAS patients.

## Supplementary information

**Additional file 1: Table S1.** Demographics of 284 patients diagnosed with metastatic angiosarcomas identified from SEER database between 2010 and 2016.

**Additional file 2: Table S2.** Median survival data (months) of metastatic angiosarcomas.

**Additional file 3: Table S3.** Univariate analysis of primary tumor sites for OS and CSS in patients of metastatic angiosarcomas.

**Additional file 4: Table S4.** The number of metastatic sites in the 284 patients diagnosed with metastatic angiosarcomas.

## Data Availability

The SEER-database is publicly available. The datasets generated and/or analyzed during the current study are also available from the corresponding author on reasonable request.

## Abbreviations

AS: Angiosarcomas
MAS: Metastatic angiosarcomas
SEER: Surveillance, Epidemiology, and End Results
OS: Overall survival
CSS: Cancer-specific survival
RT: Radiation treatment
ST: Surgery treatment
CI: Confidence interval
