# Supplementary material for: Survival predictors of metastatic angiosarcomas: a surveillance, epidemiology, and end results program population-based retrospective study
Source: BMC Cancer. 2020 Aug 18;20:778. doi: 10.1186/s12885-020-07300-7 (PMC7437028; doi:10.1186/s12885-020-07300-7)
Supplement: Supplementary file 1 — Additional file 1: Table S1. Demographics of 284 patients diagnosed with metastatic angiosarcomas identified from SEER database between 2010 and 2016. [file 12885_2020_7300_MOESM1_ESM.docx]

**Table S1.** Demographics of 284 patients diagnosed with metastatic angiosarcomas identified from SEER database between 2010 and 2016.

| **Primary tumor sites** | **N (%)** |
| --- | --- |
| Head and neck | 35(12.3%) |
| Visceral/deep soft tissue | 112(39.4%) |
| Trunk and limbs | 47(16.5%) |
| Other sites | 90(31.7%) |

**Abbreviations:** SEER, Surveillance, Epidemiology, and End Results.
