# Supplementary material for: Survival predictors of metastatic angiosarcomas: a surveillance, epidemiology, and end results program population-based retrospective study
Source: BMC Cancer. 2020 Aug 18;20:778. doi: 10.1186/s12885-020-07300-7 (PMC7437028; doi:10.1186/s12885-020-07300-7)
Supplement: Supplementary file 2 — Additional file 2: Table S2. Median survival data (months) of metastatic angiosarcomas. [file 12885_2020_7300_MOESM2_ESM.docx]

**Table S2.** Median survival data (months) of metastatic angiosarcomas.

| Primary tumor sites | OS | |  | CSS | |
| --- | --- | --- | --- | --- | --- |
|  | Estimate ± SE | 95%CI |  | Estimate ± SE | 95%CI |
| Head and neck | 7.0±1.8 | 3.470-10.530 |  | 7.0±1.8 | 3.465-10.535 |
| Visceral/deep soft tissue | 3.0±0.5 | 2.088-3.912 |  | 3.0±0.7 | 1.557-4.443 |
| Trunk and limbs | 4.0±1.2 | 1.615-6.385 |  | 3.0±1.0 | 1.051-4.949 |
| Other sites | 3.0±0.9 | 1.294-4.706 |  | 3.0±0.7 | 1.635-4.365 |

**Abbreviations:** OS, overall survival; CSS, cancer-specific survival; SE, standard error; CI, confidence interval.
