# Supplementary material for: Survival predictors of metastatic angiosarcomas: a surveillance, epidemiology, and end results program population-based retrospective study
Source: BMC Cancer. 2020 Aug 18;20:778. doi: 10.1186/s12885-020-07300-7 (PMC7437028; doi:10.1186/s12885-020-07300-7)
Supplement: Supplementary file 3 — Additional file 3: Table S3. Univariate analysis of primary tumor sites for OS and CSS in patients of metastatic angiosarcomas. [file 12885_2020_7300_MOESM3_ESM.docx]

**Table S3.** Univariate analysis of primary tumor sites for OS and CSS in patients of metastatic angiosarcomas.

| **Category** | **OS (log-rank P-value)** | **CSS (log-rank P-value)** |
| --- | --- | --- |
| Primary tumor sites | 0.162 | 0.667 |
| Head and neck **vs** visceral/deep soft tissue | 0.038 | 0.276 |
| Head and neck **vs** trunk and limbs | 0.573 | 0.726 |
| Head and neck **vs** other sites | 0.137 | 0.335 |
| Visceral/deep soft tissue **vs** trunk and limbs | 0.108 | 0.468 |
| Visceral/deep soft tissue **vs** other sites | 0.646 | 0.864 |
| Trunk and limbs **vs** other sites | 0.244 | 0.406 |

**Abbreviations:** OS, overall survival; CSS, cancer-specific survival.
