# Supplementary material for: Survival predictors of metastatic angiosarcomas: a surveillance, epidemiology, and end results program population-based retrospective study
Source: BMC Cancer. 2020 Aug 18;20:778. doi: 10.1186/s12885-020-07300-7 (PMC7437028; doi:10.1186/s12885-020-07300-7)
Supplement: Supplementary file 4 — Additional file 4: Table S4. The number of metastatic sites in the 284 patients diagnosed with metastatic angiosarcomas. [file 12885_2020_7300_MOESM4_ESM.docx]

Table S4. The number of metastatic sites in the 284 patients diagnosed with metastatic angiosarcomas.

| **The number of metastatic sites** | **N(%)** |
| --- | --- |
| Bone | 53(18.7%) |
| Brain | 9(3.2%) |
| Liver | 35(12.3%) |
| Lung | 91(32.0%) |
| Bone + brain | 3(1.1%) |
| Bone + liver | 13(4.6%) |
| Bone + lung | 32(11.3%) |
| Liver + lung | 19(6.7%) |
| Brain + lung | 7(2.5%) |
| Bone + liver + lung | 15(5.3%) |
| Brain + liver + lung | 2(0.7%) |
| Bone + brain + lung | 3(1.1%) |
| Bone + brain + liver + lung | 2(0.7%) |
| **Total** | **284(100%)** |
